# Supplementary material for: A critical assessment of the detailed Aedes aegypti simulation model Skeeter Buster 2 using field experiments of indoor insecticidal control in Iquitos, Peru
Source: PLoS Negl Trop Dis. 2022 Dec 22;16(12):e0010863. doi: 10.1371/journal.pntd.0010863 (PMC9778528; doi:10.1371/journal.pntd.0010863)
Supplement: S1 Text — (PDF) [file pntd.0010863.s001.pdf]

SI Text: Technical Details

SB2 is distributed under a GPLv3 open source license via a public GitHub repository (https://github.com/helmingstay/SkeeterBuster), and is provided without warranty or support.

SB2 input and output occurs via comma separated value (CSV) files that allow users to specify the model's parameters, initial conditions, and spatial configuration. All parameters have default values based on the best available consensus, as used in SB [1, 2]. Default values may be overridden by one or more configuration files, allowing the straightforward specification and documentation of each unique model scenario. SB2 is distributed with a suite of test scenarios that can be used to verify the proper functioning of SB2. These scenarios can also be modified by users to create novel scenarios.

Daily regional mean temperature, relative humidity, and total precipitation are provided to SB2 via a weather input file. This data can be obtained from NOAA's National Environmental Satellite, Data, and Information Service (NESDIS) Climate Data Online (CDO) portal, which provides Global Summary of the Day (GSOD) files for automated weather stations around the globe free for non-commercial use. Once an appropriate data set is located and downloaded, a set of R scripts is used to prepare the weather data for use by SB2.

The spatial structure of SB2 is specified by the relative location of houses and blocks in a list-based network that allows us to A) reproduce SB's grid-based network and B) represent the more complex geographic scenarios presented by Iquitos field data.

The spatial configurations used to simulate the Iquitos study areas were generated from a suite of GIS files maintained by the field research team as part of ongoing work in Iquitos (here, as of Jan 2016). In these, each house corresponds to a single GIS point. Zero or more houses (points) are contained within each GIS polygon. This nesting approximates the ground-truth in Iquitos, where multiple households commonly occupy individual buildings. Polygons (and their associated houses) are further grouped together in spatially-connected city blocks, which are separated by streets. Neighbor lists of houses (and blocks) were determined by geographic distance: we identified the eight nearest neighbors within 50 meters of each house, along with the four nearest blocks of each block. House distance was computed via polygon boundary, so that adjacent polygons are separated by zero distance. In addition, all houses within a polygon are separated by zero distance. Likewise, block distance was computed as the minimum distance between block boundaries. All spatial processing was done using R and PostGIS.

Container configuration and dynamics in SB2 match those of SB [1–4]. Containers in each house were specified by an initial baseline survey of that house (when one was available). When no baseline survey was available but one or more subsequent container surveys were available, a subsequent container survey was chosen at random. For the small fraction of houses with no container surveys (including houses that were never surveyed), a baseline house survey was chosen at random from both field experiments and used in place of missing surveys. While SB2 includes provisions for container movement, we do not simulate such dynamics here.

Container water level and water temperature is determined by weather data and container volume. Water level, in turn, affects the hatching of eggs within containers. Daily container food input depends on container type, volume, and location (inside vs outside). Some containers are manually emptied and refilled, which removes all immature insects and accumulated food from the container. Adult and immature insects' maturation is specified by an enzyme kinetics sub-model [5], whose rate is determined by air temperature and container water temperature, respectively. SB employs a model time step of one day, except for the enzyme kinetics sub-model, which is evaluated at a 3-hour timestep (8 steps per day).

SB2 includes the insect genetics sub-modules provided by SB. We have not considered insect genetics here, though we note that the spatial spread and fixation of insecticide resistance genes amongst an *Ae. aegypti* population remains a compelling example to further explore with SB2.

References

[1] Krisztian Magori, Mathieu Legros, Molly E Puente, Dana A Focks, Thomas W Scott, Alun L Lloyd, and Fred Gould. Skeeter Buster: A Stochastic, Spatially Explicit Modeling Tool for Studying *Aedes aegypti* Population Replacement and Population Suppression Strategies. *PLoS Neglected Tropical Diseases*, 3(9):e508, 2009.

[2] Mathieu Legros, Krisztian Magori, Amy C Morrison, Chonggang Xu, Thomas W Scott, Alun L Lloyd, and Fred Gould. Evaluation of location-specific predictions by a detailed simulation model of *Aedes aegypti* populations. *PLOS ONE*, 6(7):e22701, 2011.

[3] Amy C Morrison, Kenneth Gray, Arthur Getis, Helvio Astete, Moises Sihuincha, Dana Focks, Douglas Watts, Jeffrey D Stancil, James G Olson, Patrick Blair, et al. Temporal and geographic patterns of *Aedes aegypti* (diptera: Culicidae) production in iquitos, peru. *Journal of medical entomology*, 41(6):1123–1142, 2004.

[4] Jennifer R Schneider, Amy C Morrison, Helvio Astete, Thomas W Scott, and Mark L Wilson. Adult size and distribution of *Aedes aegypti* (diptera: Culicidae) associated with larval habitats in iquitos, peru. *Journal of medical entomology*, 41(4):634–642, 2004.

[5] D Ae Focks, DG Haile, E Daniels, and G Ae Mount. Dynamic life table model for *Aedes aegypti* (diptera: Culicidae): analysis of the literature and model development. *Journal of Medical Entomology*, 30(6):1003–1017, 1993.
