# Supplementary material for: A critical assessment of the detailed Aedes aegypti simulation model Skeeter Buster 2 using field experiments of indoor insecticidal control in Iquitos, Peru
Source: PLoS Negl Trop Dis. 2022 Dec 22;16(12):e0010863. doi: 10.1371/journal.pntd.0010863 (PMC9778528; doi:10.1371/journal.pntd.0010863)
Supplement: S1 Table — When available, baseline surveys were used to parameterize each house’s container configuration. (PDF) [file pntd.0010863.s002.pdf]

Supplemental Tables and Figures for Gunning et al. 2022

Assessing a detailed Aedes aegypti simulation model using field control experiments.

Table S1: House counts in the baseline circuit (C1) of each experiment, showing the proportion of houses with and without successful surveys. When available, baseline surveys were used to parameterize each house's container configuration.

Experiment	Survey	Houses	Proportion
S-2013	Yes	943	0.719
S-2013	No	368	0.281
L-2014	Yes	1470	0.659
L-2014	No	762	0.341
