# Supplementary material for: A critical assessment of the detailed Aedes aegypti simulation model Skeeter Buster 2 using field experiments of indoor insecticidal control in Iquitos, Peru
Source: PLoS Negl Trop Dis. 2022 Dec 22;16(12):e0010863. doi: 10.1371/journal.pntd.0010863 (PMC9778528; doi:10.1371/journal.pntd.0010863)
Supplement: S2 Table — Dispersal parameters show per-day probabilites. Container food input multiplier was determined by matching mean adult populations in the buffer sector between model and data (including both experiments together). Spray efficacy was empirically determined for each experiment from observed cage mortality: S-2013 = 0.91; L-2014 = 0.72. Finally, spray efficacy was systematically varied from the reference scenario (Low = 0.5; High = 1). (PDF) [file pntd.0010863.s003.pdf]

Table S2: Key parameters of default scenario. Dispersal parameters show per-day probabilites. Container food input multiplier was determined by matching mean adult populations in the buffer sector between model and data (including both experiments together). Spray efficacy was empirically determined for each experiment from observed cage mortality: S-2013=0.91; L-2014=0.72. Finally, spray efficacy was systematically varied from the default scenario (Low=0.5; High=1).

Parameter	Value
nulliparous_female_adult_long_range_dispersal	0.001
parous_female_adult_long_range_dispersal	0.001
male_adult_long_range_dispersal	0.001
container_movement_probability	0
nulliparous_female_adult_dispersal	0.1
parous_female_adult_dispersal	0.1
male_adult_dispersal	0.1
nulliparous_female_adult_dispersal_from_empty_house	0.1
parous_female_adult_dispersal_from_empty_house	0.1
male_adult_dispersal_when_no_female	0.1
do_adult_spraying	TRUE
do_cont_sampling	TRUE
do_adult_sampling	TRUE
proportion_of_adult_females_sampled	0.29
proportion_of_adult_males_sampled	0.29
food_input_multiplier	0.65
