# Supplementary material for: A critical assessment of the detailed Aedes aegypti simulation model Skeeter Buster 2 using field experiments of indoor insecticidal control in Iquitos, Peru
Source: PLoS Negl Trop Dis. 2022 Dec 22;16(12):e0010863. doi: 10.1371/journal.pntd.0010863 (PMC9778528; doi:10.1371/journal.pntd.0010863)
Supplement: S3 Table — Values show ensemble summary (reference scenario): median 95% PI (empirical). * denotes spray circuits. See also Fig 7 and S4 Table. AA/HSE: Ae. aegypti adults per house (sampled). AHI: Adult House Index. PrNF: Sample proportion nulliparous females. PC/HSE: Positive containers per house (sampled). (PDF) [file pntd.0010863.s004.pdf]

Table S3. Ratio of sector means (spray / buffer) by circuit, as in Fig 7. Values show ensemble summary (reference scenario): **median** 95% PI (empirical). * denotes spray circuits. See also Fig 7 and S4 Table. **AA/HSE**: *Ae. aegypti* adults per house (sampled). **AHI**: Adult House Index. **PrNF**: Sample proportion nulliparous females. **PC/HSE**: Positive containers per house (sampled).

Exper.	Circuit	AA/HSE	AHI	PrNF	PC/HSE
S-2013	C1	**0.89** 0.77-1 (1.5)	**0.95** 0.82-1.1 (1.1)	**1.1** 0.91-1.3 (2)	**0.95** 0.92-0.97 (0.98)
	C2 *	**0.53** 0.4-0.66 (0.26)	**0.62** 0.49-0.79 (0.38)	**1.7** 1.4-2.1 (0.95)	-
	C3	**0.37** 0.28-0.46 (0.41)	**0.46** 0.37-0.57 (0.6)	**1.1** 0.88-1.4 (0.33)	**0.49** 0.42-0.56 (0.41)
	C4	**0.56** 0.4-0.75 (0.84)	**0.62** 0.47-0.81 (0.74)	**1** 0.82-1.3 (1.1)	**0.57** 0.45-0.67 (1.2)
L-2014	C1	**0.94** 0.85-1.1 (1.2)	**0.95** 0.87-1 (1.1)	**1.1** 0.86-1.3 (2)	**0.97** 0.93-1 (1)
	C2	**0.9** 0.61-1.3 (0.9)	**0.85** 0.59-1.2 (0.89)	**1.2** 0.66-2.2 (0.088)	-
	C3 *	**0.94** 0.7-1.3 (0.82)	**0.96** 0.72-1.4 (1.1)	**1.1** 0.81-1.5 (1.1)	-
	C4	**0.9** 0.78-1.1 (0.91)	**0.93** 0.78-1 (0.88)	**0.98** 0.81-1.2 (1.7)	**0.9** 0.84-0.96 (1.1)
	C5	**1** 0.81-1.3 (1.5)	**0.98** 0.82-1.2 (1.3)	**1** 0.82-1.2 (1.4)	-
	C6 *	**0.57** 0.47-0.67 (0.67)	**0.61** 0.52-0.71 (0.52)	**1.4** 1.2-1.5 (3.5)	-
	C7	**0.57** 0.46-0.67 (0.71)	**0.63** 0.51-0.74 (0.82)	**1** 0.82-1.3 (1.3)	**0.64** 0.58-0.71 (0.82)
	C8	**0.81** 0.7-0.97 (1.9)	**0.79** 0.67-0.92 (1.5)	**1** 0.87-1.3 (1)	**0.85** 0.78-0.94 (1)
	C9	**0.91** 0.74-1.1 (2.5)	**0.86** 0.73-1 (1.6)	**1** 0.8-1.2 (3.7)	**0.88** 0.81-0.97 (1.6)
