# Supplementary material for: A critical assessment of the detailed Aedes aegypti simulation model Skeeter Buster 2 using field experiments of indoor insecticidal control in Iquitos, Peru
Source: PLoS Negl Trop Dis. 2022 Dec 22;16(12):e0010863. doi: 10.1371/journal.pntd.0010863 (PMC9778528; doi:10.1371/journal.pntd.0010863)
Supplement: S4 Table — See also Fig 7. AA/HSE: Ae. aegypti adults per house (sampled). AHI: Adult House Index. PrNF: Sample proportion nulliparous females. PC/HSE: Positive containers per house (sampled). (PDF) [file pntd.0010863.s005.pdf]

Table S4. Ratio of circuit means (indicated circuit / baseline) within the spray sector (values as in S3 Table). See also Fig 7. **AA/HSE**: *Ae. aegypti* adults per house (sampled). **AHI**: Adult House Index. **PrNF**: Sample proportion nulliparous females. **PC/HSE**: Positive containers per house (sampled).

Exper.	Circuit	AA/HSE	AHI	PrNF	PC/HSE
S-2013	C2 *	**0.25** 0.19-0.31 (0.17)	**0.38** 0.3-0.46 (0.35)	**1.6** 1.3-1.9 (0.4)	-
	C3	**0.31** 0.24-0.38 (0.55)	**0.4** 0.32-0.48 (0.79)	**1.1** 0.91-1.5 (0.16)	**0.45** 0.39-0.52 (0.75)
	C4	**0.27** 0.2-0.36 (0.89)	**0.36** 0.28-0.47 (1.1)	**1.2** 0.92-1.6 (0.79)	**0.49** 0.4-0.57 (2)
L-2014	C2	**0.43** 0.31-0.61 (0.85)	**0.52** 0.4-0.67 (0.86)	**0.91** 0.62-1.3 (0.43)	-
	C3 *	**0.3** 0.23-0.38 (0.39)	**0.39** 0.31-0.48 (0.54)	**1.4** 1.1-1.8 (1.6)	-
	C4	**0.56** 0.47-0.67 (0.69)	**0.64** 0.55-0.72 (0.69)	**1.3** 1.1-1.6 (1.7)	**0.64** 0.59-0.67 (0.85)
	C5	**0.66** 0.55-0.79 (0.88)	**0.71** 0.61-0.82 (0.83)	**1.2** 0.96-1.4 (1.3)	-
	C6 *	**0.43** 0.37-0.49 (0.4)	**0.49** 0.43-0.55 (0.33)	**1.6** 1.4-1.8 (3)	-
	C7	**0.48** 0.4-0.56 (0.67)	**0.53** 0.45-0.62 (0.66)	**1.1** 0.88-1.3 (1.5)	**0.49** 0.45-0.53 (1.2)
	C8	**0.59** 0.51-0.71 (1.5)	**0.62** 0.54-0.72 (1)	**1.2** 1-1.4 (0.69)	**0.59** 0.55-0.64 (0.59)
	C9	**0.82** 0.67-0.95 (1.7)	**0.74** 0.66-0.89 (1.2)	**1.1** 0.86-1.3 (1.6)	**0.71** 0.66-0.76 (0.69)
