# Supplementary material for: A critical assessment of the detailed Aedes aegypti simulation model Skeeter Buster 2 using field experiments of indoor insecticidal control in Iquitos, Peru
Source: PLoS Negl Trop Dis. 2022 Dec 22;16(12):e0010863. doi: 10.1371/journal.pntd.0010863 (PMC9778528; doi:10.1371/journal.pntd.0010863)
Supplement: S1 Fig — See also Fig 2. (PDF) [file pntd.0010863.s006.pdf]

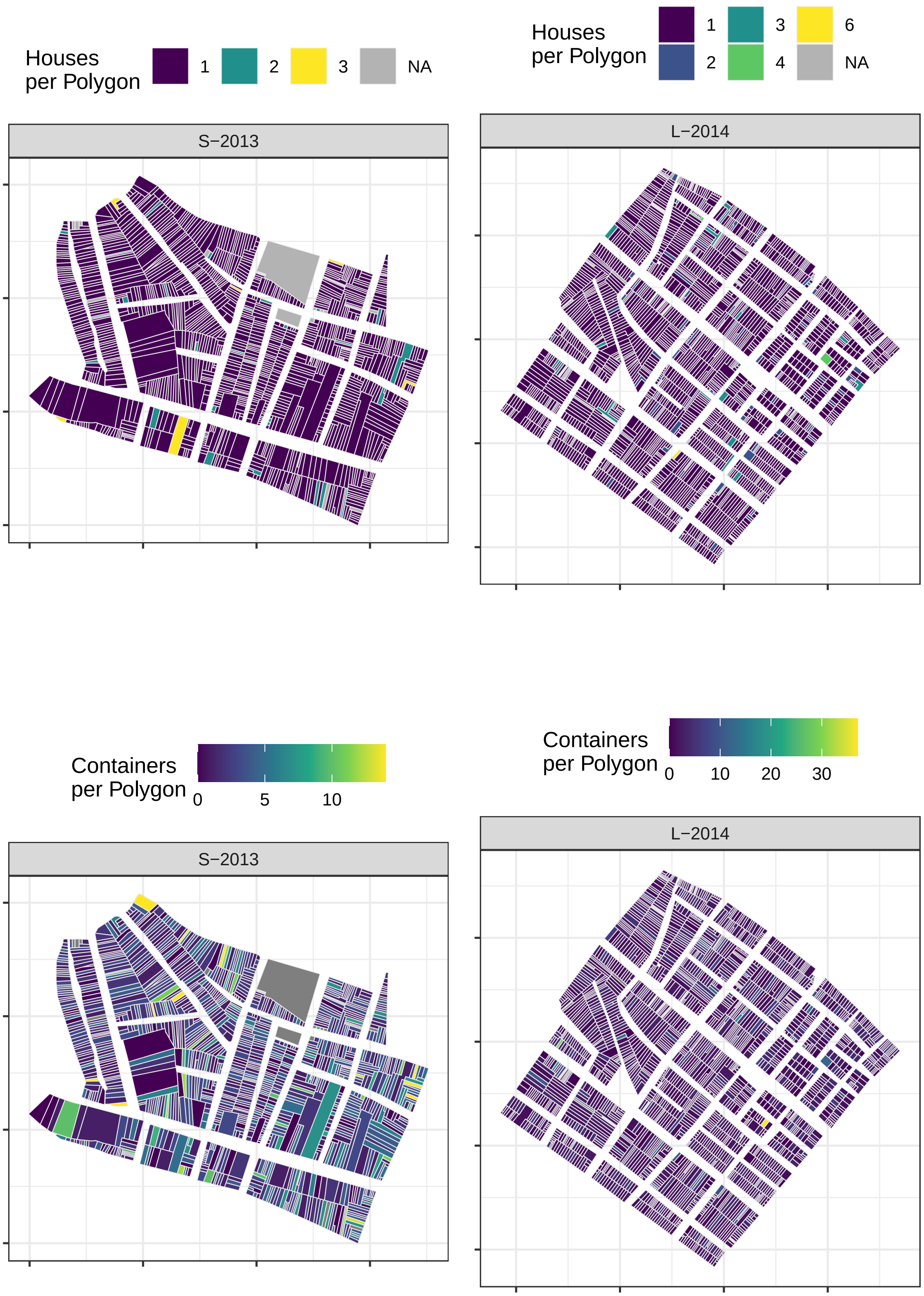

Figure S1: Map of simulation configuration, showing houses per polygon (top row) and containers per polygon (bottom row). See also Figure 2.
