# Supplementary material for: A critical assessment of the detailed Aedes aegypti simulation model Skeeter Buster 2 using field experiments of indoor insecticidal control in Iquitos, Peru
Source: PLoS Negl Trop Dis. 2022 Dec 22;16(12):e0010863. doi: 10.1371/journal.pntd.0010863 (PMC9778528; doi:10.1371/journal.pntd.0010863)
Supplement: S2 Fig — Vertical dashed lines show spray events (see Fig 3). (PDF) [file pntd.0010863.s007.pdf]

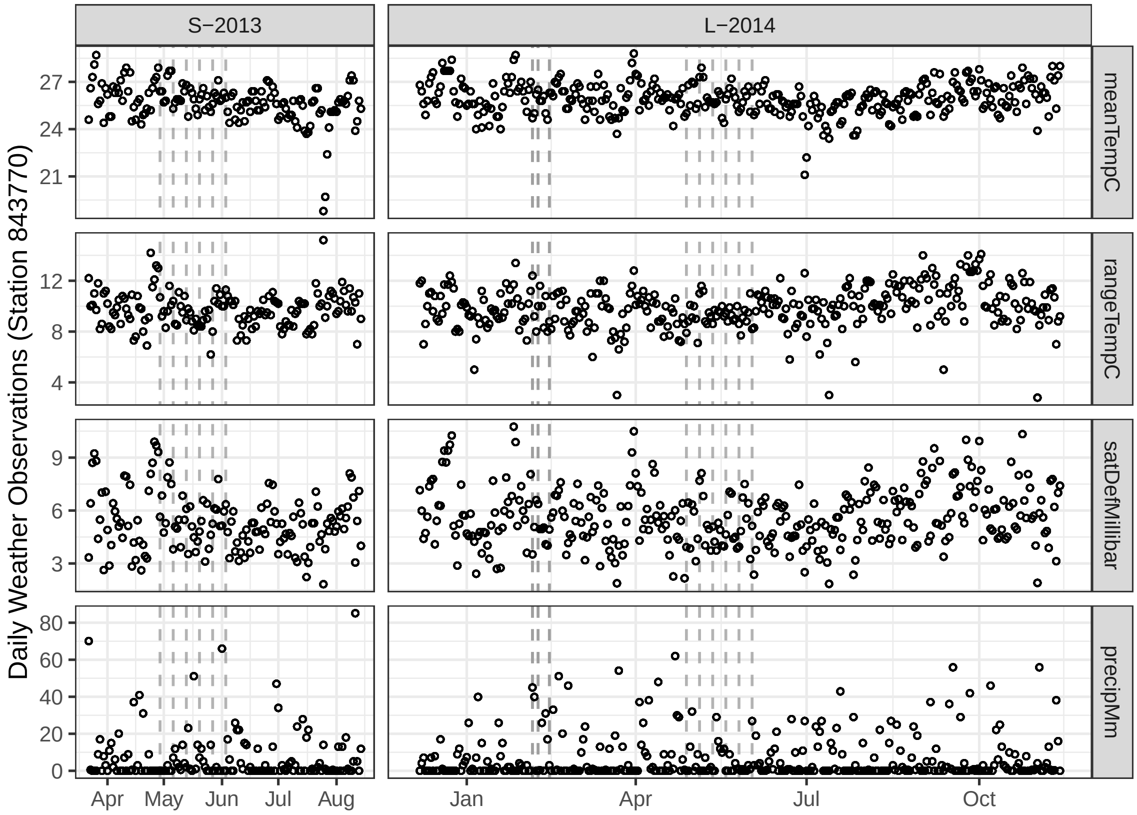

Figure S2: Daily time series of observed weather at station 843770. Vertical dashed lines show spray events (see Figure 3).
