# Supplementary material for: A critical assessment of the detailed Aedes aegypti simulation model Skeeter Buster 2 using field experiments of indoor insecticidal control in Iquitos, Peru
Source: PLoS Negl Trop Dis. 2022 Dec 22;16(12):e0010863. doi: 10.1371/journal.pntd.0010863 (PMC9778528; doi:10.1371/journal.pntd.0010863)
Supplement: S3 Fig — Adult females closely track total adult populations, while eggs and larvae have an inverse response as eggs transition into larvae. In the short-term (<5 days), increased temperature and humidity yield increases in adults and eggs, and a decrease in proportion nulliparous females; precipitation causes eggs to hatch into larvae, while increased temperatures cause larvae to develop into pupae. High humidity is also associated with lower larval populations across a range of time lags, most strongly at <10 days. In the medium to long-term (10-20 days), increased temperature and humidity was associated with increased pupal populations (peak correlation at approx. 10 days), while precipitation was associated with increased adult populations (peak correlation at approx. 18 days). No consistent correlation between weather and number of positive containers per house is evident. (PDF) [file pntd.0010863.s008.pdf]

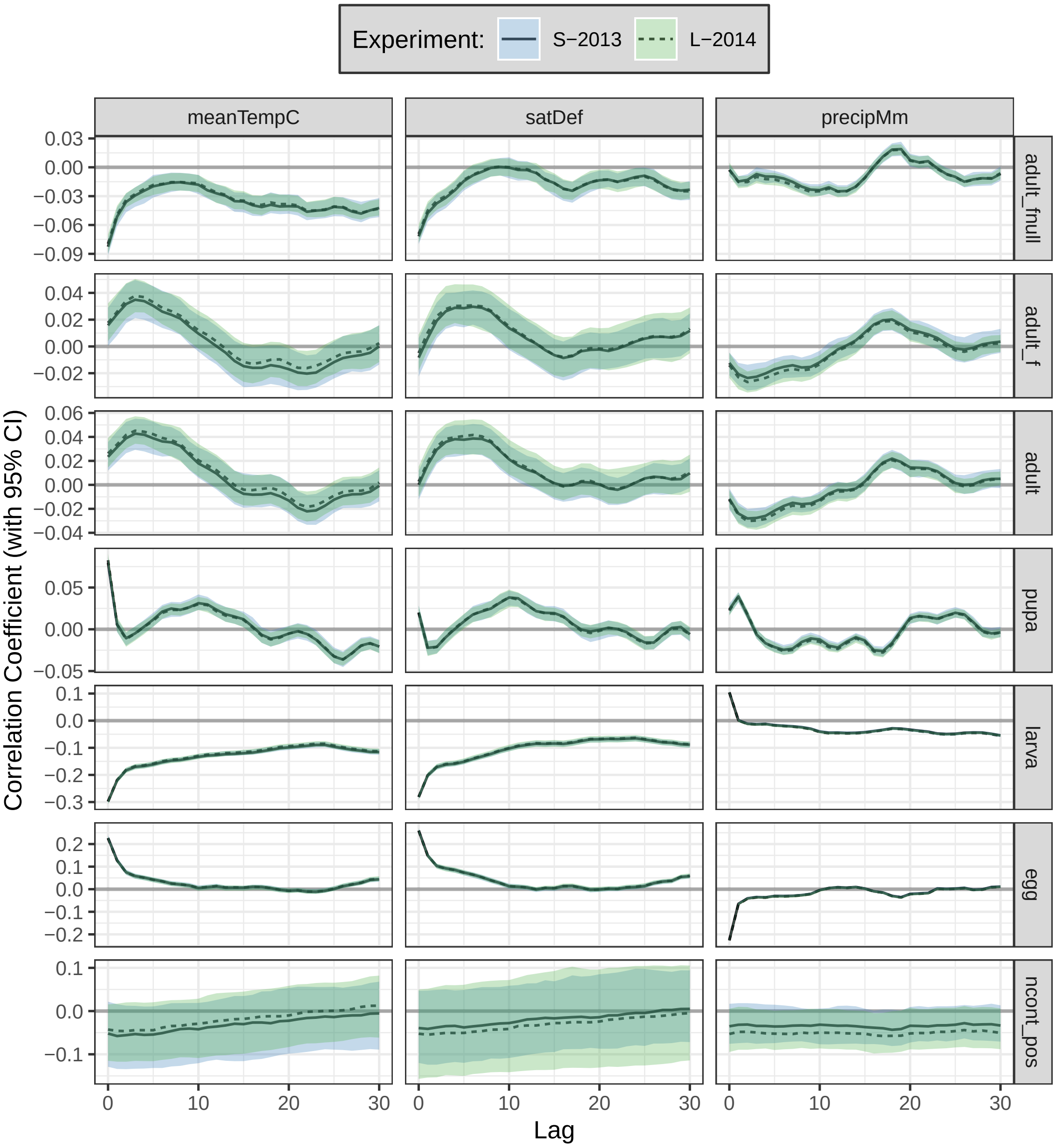

Figure S3: Correlation between weather and insect populations at different life stages for long-running scenario. Adult females closely track total adult populations, while eggs and larvae have an inverse response as eggs transition into larvae. In the short-term (<5 days), increased temperature and humidity yield increases in adults and eggs, and a decrease in proportion nulliparous females; precipitation causes eggs to hatch into larvae, while increased temperatures cause larvae to develop into pupae. High humidity is also associated with lower larval populations across a range of time lags, most strongly at <10 days. In the medium to long-term (10-20 days), increased temperature and humidity was associated with increased pupal populations (peak correlation at approx. 10 days), while precipitation was associated with increased adult populations (peak correlation at approx. 18 days). No consistent correlation between weather and number of positive containers per house is evident.
