# Supplementary figures and images for: A critical assessment of the detailed Aedes aegypti simulation model Skeeter Buster 2 using field experiments of indoor insecticidal control in Iquitos, Peru
Source: PLoS Negl Trop Dis. 2022 Dec 22;16(12):e0010863. doi: 10.1371/journal.pntd.0010863 (PMC9778528; doi:10.1371/journal.pntd.0010863)

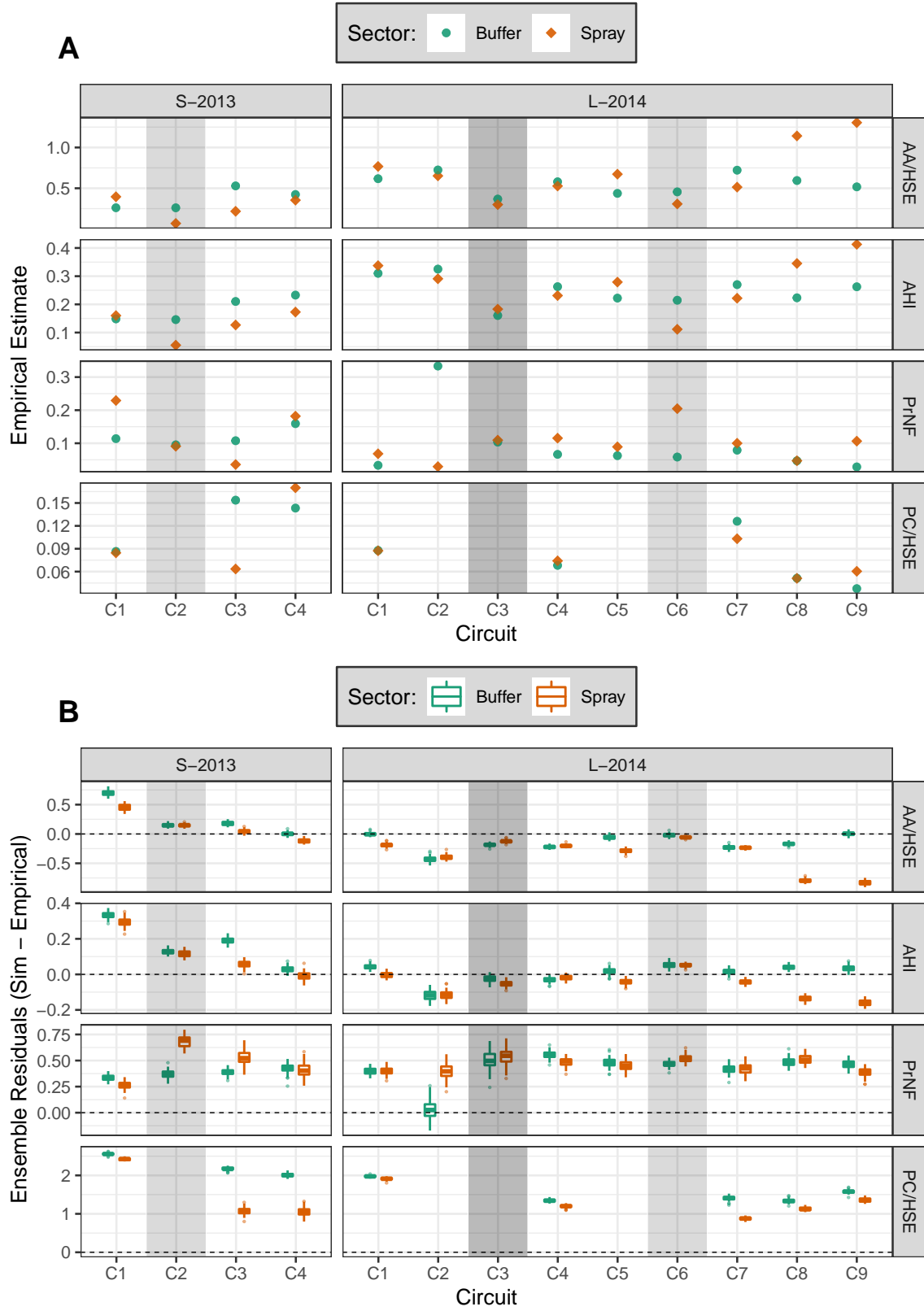

Supplement: S4 Fig — A: empirical results (as in Gunning et al. [32]). B, ensemble residuals (simulated—empirical). See also Fig 6. AA/HSE: Ae. aegypti adults per house (sampled). AHI: Adult House Index. PrNF: Sample proportion nulliparous females. PC/HSE: Positive containers per house (sampled). (PDF) [file pntd.0010863.s009.pdf]
