# Supplementary material for: A critical assessment of the detailed Aedes aegypti simulation model Skeeter Buster 2 using field experiments of indoor insecticidal control in Iquitos, Peru
Source: PLoS Negl Trop Dis. 2022 Dec 22;16(12):e0010863. doi: 10.1371/journal.pntd.0010863 (PMC9778528; doi:10.1371/journal.pntd.0010863)
Supplement: Attachment — Submitted filename: gunning-response.pdf [file pntd.0010863.s010.pdf]

Response to Reviewers

C.E. Gunning et al., April 1, 2022. *A critical assessment of the detailed Aedes aegypti simulation model Skeeter Buster 2 using field experiments of indoor insecticidal control in Iquitos, Peru.*

Overview

We appreciate the reviewers detailed attention to our work. In light of their comments, we have revised the text to clarify our aims and goals, improve organization, provide important details in appropriate locations, and improve the quantitative display of results. We believe these revisions substantially improve the overall presentation and impact of our work. We provide detailed responses to specific comments below.

Editorial and Data Presentation Modifications

Reviewer 2

1. Can you specify the insecticide used in ULV control?

We have revised our Methods (*Spray Intervention*) to include additional detail regarding spray applications that includes pyrethroid formulation, application equipment, and quality control. We have attempted throughout to streamline the experimental details of this manuscript, and to refer readers to the appropriate sections of the preceding empirical paper that describes the field methods in extensive detail [1].

2. How confident can you be that Prokopack aspirators collect an unbiased sample of adult female mosquitoes, with respect to their parity? Depending on their behaviour (e.g. mate-seeking, blood-seeking, resting, ovipositing etc), one might expect females are more or less readily caught by any particular capture method. If there is such a bias, this may affect the empirical estimation of nulliparous frequency – did the authors consider this? It would be helpful for this to be commented in the methods section.

We agree with the importance of this question, given the potential impact of Æ female age distribution on key ecological and epidemiological processes. We have revised the relevant section of discussion to highlight the empirical uncertainty and potential bias based on differential behavior.

In terms of sampling, we have no reason to expect a strong bias in adult female collection in houses with respect to parity. To our knowledge, the best empirical evidence of this comes from Koyoc-Cardeña et al. [2], who tested exhaustive sampling of houses in Merida, Mexico. They note that "sensitivity of detection of positive houses in the first sampling round was 82.5% for any adult Ae. aegypti, 78.5% for females, 75.5% for males and 73.3% for blood-fed females" (see also Fig. 2 there).

We note that our field experiments were not designed to detect differential behavior in Æwith respect to parity around, for example, house occupancy. We also previously stated (*Discussion, Female Age Distribution*) that empirical parity assessments relied on dissection and visual scoring that "constitute a potential source of error", with observed variation among observers. In this regard, our parity results mirror our discussion of cryptic larval habitat, where the import "known unknowns" remains difficult to assess without intensive field efforts. We have also revised the methods to clarify that adult collections occurred A) within and around surveyed houses and B) included active searches by trained workers.

3. In the "Simulation Overview" section, a number of functional relationships embedded in the model are alluded to: "Adult movement depends on the availability of mates and larval habitat (we assume human hosts are available in all houses)... oviposition choice depends on container volume and the presence of a cover or lid, and larval development depends on weather and food availability. Survival of all life stages depends on temperature. Survival of adults and eggs also depends on water vapor pressure deficit, which is a function of temperature and relative humidity". As a modeller but without any experience with this particular model, I wanted to know more about these assumed functional relationships. I would have particularly appreciated a figure (supplementary if the authors do not want more main manuscript figures) showing the shapes of the relationships. Having them explained in words in the section here would also be helpful.

With several key exceptions (noted in *Methods, Simulation Overview*), SB2 remains identical to SB in its representation of Æ biology. Consequently, we have attempted to balance the detail provided here with the extensive body of existing literature on the SB model [3–6], along with its non-spatial deterministic predecessor CIMSiM [7]. Unfortunately, a straightforward diagram of functional relationships between the noted elements is complicated by a range of non-linear interactions. In total, the previously-described governing equations (and the implementing model source code) are the most reliable description of our model. Fortunately, our model code is now hosted in a public GitHub repository and distributed under a GPL license for future inspection and re-use.

We have revised this section to more clearly highlight the relevant literature (particularly [3] Figs 1-5), provide more detail on certain functional relationships, and note the availability and location of the source code. We also note that Figure S3 visualizes the observed functional response of each life stage to variations in temperature, humidity, and precipitation across a long-running scenario.

4. You might say something about how easy or otherwise it would be to apply the model to other settings. We have revised the manuscript to highlight existing comparisons between SB in Iquitos versus Buenos Aires (Legros et al. [5] and Legros et al. [6]), and have added details (*Methods, Simulation Overview*) about the use and availability of location-specific weather data. We have also revised the conclusion to better motivate our focus on Iquitos, and to better set the current work in a global context.

Summary and General Comments

Reviewer 1

Generally, the statistical procedures for model fitting and validation could be improved. Currently the model is calibrated to the data via a single multiplicative parameter (container food availability) and model fit evaluated descriptively and visually (Fig. 7-8). I understand the authors intentions about minimally modifying the model to retain generalisability, but really think that both of these should be improved. On fitting/calibration ideally multiple parameters should be selected and justified for model fitting, then a formal model fitting exercise be performed (even a simple parameter sweep within ranges suggested by previous datapoints would give more insights into the identifiability of the model when fit to data).

We appreciate this comment, which highlights that we did not clearly enunciate the goals of our manuscript from the outset. The focus of our paper is model validation, and we have intentionally refrained from model *fitting*. We note at several points that our model was previously parameterized (i.e., fit) using experimental data from Iquitos under equilibrium conditions. A primary goal of this study is to test the ability of this model (and its previously-ascertained fit) to reproduce out-of-sample empirical observations. In this case, we have "tuned" a single model parameter (food input) to match observed mean abundance, as our focus is on the dynamical response to perturbation rather than absolute mosquito abundance (which is, in itself, challenging to reliably ascertain).

In our original submission, a detailed discussion of this important distinction was not provided until the discussion (*Model Validation*). We have revised our introduction to highlight our core objectives at the outset. We have also clarified here the relationship between SB and SB2, and our reliance on SB's parameterization in Iquitos.

I also think there should be some form of statistical validation of the model fit to the empirical data. This is needed to quantify the weight of evidence behind the currently suggested reasons for discrepancy between simulated and observed data and would provide a framework for the hypothesis testing that the authors claim this model will enable.

While we agree with the spirit of the comment, we note several important points. First, while multiple modes of variability exist simultaneously within this study, we have chosen to focus on A) variation among realizations within the ensemble of B) the mean response of key observables (AA/HSE, AHI, PrNF, and PC/HSE) across space and time. This choice is largely motivated by a desire for brevity and clarity in the face of complexity. For example, the confidence intervals in Gunning et al. [1] (e.g., Figs 4-5) denote empirical observational uncertainty estimated from theoretically motivated parametric sampling distributions. In the present work, the key figures comparing simulation results with empirical data (Figs 7-8) show 95% prediction intervals that represent stochasticity *between* simulations. To avoid confusion, we have opted to exclude empirical uncertainty in Figs 7-8, since these two sources of variation are not comparable (we also believe that a multi-tiered comparison of within- and among-simulation variation is outside

the scope of the current work).

In our key comparison figures (Figs 7-8), we shown 95% prediction intervals based on model ensembles. In this way, we have attempted to transparently present simulation results in a way that allows readers to explore comparisons of particular interest. Given the non-linear dynamics of these observables, we believe that global parametric statistical test would provide a misleading view of our results. On the other hand, simple non-parametric tests are precluded by the SB2's noteworthy failure to reproduce certain dynamics, e.g., during L-2014 C8-9.

I was surprised about the magnitude of the impact of destructive container sampling (Fig. 4) until I read about the assumption of no cryptic breeding sites. This seems like an assumption that is at least worth some sensitivity analysis as I find it difficult to believe that 100% of containers can be accurately identified and it would improve our understanding of the dynamics of this model if we could explore its sensitivity to relaxing this assumption.

We agree that this is an important point, as well as a somewhat neglected and empirically challenging area of Æ population biology with important epidemiological consequences. We have revised the manuscript results to clearly highlight earlier in the manuscript our omission of cryptic habitat.

We note that our simulations incorporate the empirically observed location, size, and type of larval habitat. As we have not directly observed cryptic habitat in the study, we do not include it in our simulations. Given the spatially explicit structure of SB2, including cryptic habitat in the current study would require nontrivial assumptions about the locations and specific configuration of such habitat, and we expect that the proposed analysis would be sensitive to these assumptions. While important, we believe that a detailed analysis of cryptic larval habitat is outside the scope of the current manuscript. We have revised the methods and results to more clearly highlight this key limitation.

Minor comments: The figures are difficult to interpret in isolation and some changes could be made to the legend or figure text to improve interpretability, e.g. define acronyms in the legend text. Also y-axis labels for figures 1-5- what does "Adults" (range 1-3) refer to- adults per some unit of area, relative to some particular timepoint?

Thank you for these detailed comments. We have revised the captions in Figs 3-9 for clarity. For model dynamics (Figs 3-5), we have included detailed information in the Figs 3 caption, while the captions of Figs 4-5 refer readers to Fig 3 for details. We have also updated axis labels in Figs 3-5 for clarity, and added the full list of relevant abbreviations to Figs 7-9 as well as appropriate supplemental figures and tables.

Reviewer 2

Though numerous models of mosquito vectors of disease have been published in recent years, few have been compared against real data. In making such a comparison, this study is useful for two reasons. First, there is an immediate benefit to validating the specific model, called SB2, giving confidence in its ability to provide insight for programmes of Aedes aegypti control. Second, the study provides a lesson in the potential, and also challenges, of validating mosquito population models with data.

We appreciate the reviewer's enthusiasm, and agree with their assessments of both the rarity and specific usefulness of our study.

References

[1] Christian E Gunning, Kenichi W Okamoto, Helvio Astete, Gissella M Vasquez, Erik Erhardt, Clara Del Aguila, Raul Pinedo, Roldan Cardenas, Carlos Pacheco, Enrique Chalco, et al. Efficacy of *Aedes aegypti* control by indoor ultra low volume (ulv) insecticide spraying in iquitos, peru. *PLoS Neglected Tropical Diseases*, 12(4):e0006378, 2018.

[2] Edgar Koyoc-Cardeña, Anuar Medina-Barreiro, Azael Cohuo-Rodríguez, Norma Pavía-Ruz, Audrey Lenhart, Guadalupe Ayora-Talavera, Mike Dunbar, Pablo Manrique-Saide, and Gonzalo Vazquez-Prokopec. Estimating absolute indoor density of *Aedes aegypti* using removal sampling. *Parasites & vectors*, 12(1):1–11, 2019.

[3] Krisztian Magori, Mathieu Legros, Molly E Puente, Dana A Focks, Thomas W Scott, Alun L Lloyd, and Fred Gould. Skeeter Buster: A Stochastic, Spatially Explicit Modeling Tool for Studying *Aedes aegypti* Population Replacement and Population Suppression Strategies. *PLoS Neglected Tropical Diseases*, 3(9):e508, 2009.

[4] Chonggang Xu, Mathieu Legros, Fred Gould, and Alun L Lloyd. Understanding Uncertainties in Model-Based Predictions of *Aedes aegypti* Population Dynamics. *PLoS Neglected Tropical Diseases*, 4(9):e830, 2010.

[5] Mathieu Legros, Krisztian Magori, Amy C Morrison, Chonggang Xu, Thomas W Scott, Alun L Lloyd, and Fred Gould. Evaluation of location-specific predictions by a detailed simulation model of *Aedes aegypti* populations. *PLOS ONE*, 6(7): e22701, 2011.

[6] Mathieu Legros, Marcelo Otero, Victoria Romeo Aznar, Hernan Solari, Fred Gould, and Alun L Lloyd. Comparison of two detailed models of *Aedes aegypti* population dynamics. *Ecosphere*, 7(10):e01515, 2016.

[7] D Ae Focks, DG Haile, E Daniels, and G Ae Mount. Dynamic life table model for *Aedes aegypti* (diptera: Culicidae): analysis of the literature and model development. *Journal of Medical Entomology*, 30(6):1003–1017, 1993.
