# Supplementary material for: A critical assessment of the detailed Aedes aegypti simulation model Skeeter Buster 2 using field experiments of indoor insecticidal control in Iquitos, Peru
Source: PLoS Negl Trop Dis. 2022 Dec 22;16(12):e0010863. doi: 10.1371/journal.pntd.0010863 (PMC9778528; doi:10.1371/journal.pntd.0010863)
Supplement: Attachment — Submitted filename: gunning-response.2022-08.pdf [file pntd.0010863.s011.pdf]

Response to suggested changes for the manuscript *A critical assessment . . .*

Gunning et al., August 2022

We again thanks the reviewers and editor for their careful attention to detail. We have updated the manuscript to incorporate the suggested changes. Please see response to specific comments below.

Summary.

> The Aedes aegypti mosquito is commonly found in tropical urban areas, and is the primary vector of several serious human pathogens, including the yellow fever, Zika, and dengue viruses.

consider removing 'the' preceding Aedes aegypti and yellow fever for readers in the PLoSNTD audience.

Fixed.

Methods.

> Figure 1: Map showing study areas within Iquitos, Peru. Color shows sector. The larger, northern L-2014 study area borders an abandon air strip on its northwest edge. Base map by Stamen Design, under CC BY 3.0; data by OpenStreetMap, under ODbL. *Please add to Figure legend to indicate years when spray event experiments were conducted at each site, and edit spelling error for 'abandon'*

Fixed.

Field Studies.

> "We enumerated the number of adult mosquitoes per house by species. In addition, the parity of adult Ae. aegypti females was assessed post-collection by dissection and inspection of ovaries."

Please provide a reference to methods for parity assessment after this sentence, in the interest of enhancing the repeatability of your work and making it accessible to researchers who have not used this method:

Added.

Spray Intervention.

> During scheduled experimental spraying, small screened cages, each containing 25 adult Ae. aegypti from a recently collected laboratory colony (Gunning 2018)

please specify sex of adult mosquitoes placed in screened cages.

We have confirmed that these are females; fixed.

> When mortality was ¡80%,

note unnecessary punctuation here.

Latex error, fixed.

Simulation Overview.

> In brief, SB and SB2 model individual adult female Ae. aegypti, and cohorts of adult males and those of immature stages: eggs, larvae, and pupae. Adult Ae. aegypti mosquitoes (henceforth adults) dwell within and move among houses, which contain larval habitat (containers). Adult movement depends on the availability of mates and larval habitat (we assume human hosts are available in all houses). Adult females mate and subsequently lay eggs in water-filled containers, where container oviposition choice depends on container volume and the presence of a cover or lid, and larval survival development depends on food availability. Survival of all life stages depends on temperature. Survival of adults and eggs also depends on water vapor pressure deficit (VPD, a function of temperature and relative humidity), with mortality increasing at low VPD. Finally, SB2 models per-house destructive sampling of adults and immature insects within containers (i.e., eggs and larvae), as well as the impacts of indoor ULV spraying on adults

»In this section, it would be helpful to provide references for the life history traits described herein, with particular reference to data from Ae. aegypti in Iquitos.

We have substantially revised and reordered this section to clearly indicate the extensive body of previous work regarding SB design and parameterization, and to more clearly indicate that we made no key changes to SB here.

We note that our principle intent in this section is to offer a high-level overview to aid interpretation of the results. We also note that detailed site-specific field data regarding life history traits remains scarce.

> See sentence "We note that, while SB and SB2 were parameterized using field data from Iquitos, its performance has also been evaluated in Buenos Aires, Argentina <insert 'by'> Legros et al. [19, 23]."

»missing word or parenthesis - please see <> above

Fixed, thank you

»Please check that Table 1 is as intended. The legend indicates that the table is a summary of the model configuration, but it appears to be a summary of the data used, and does not appear to speak to other elements of the caption as provided. The table is more correctly described in text, which reads "A summary of each field experiment's configuration, including total number of houses and containers, containers per house, and container-free houses by sector, is shown in Table 1 (see also Table S1)."

Thank you. We have revised both the text and the caption to more clearly

indicate that the table shows simulation configuration, which is generated from field observations.

Results.

Simulation Dynamics.

»Please check references to figures to be sure that the text connects to and describes each Figure. For example, the text reads "The SB2 reference scenario was developed to simulate the S-2013 and L-2014 field experiments as closely as possible. Our simulations incorporate the spatial location of houses and their containers, as well as the observed location and timing of empirical field sampling and spraying (Figure 3)." I note that the title for Figure 3 is "Figure 3: Daily time series of model dynamics (not survey results) grouped by sector (color) for the default scenario" and that this isn't well aligned with the text.

We have revised this text.

> Figure 3: Daily time series of model dynamics (not survey results) grouped by sector (color) for the default scenario

»Please provide more information about "the default scenario" to make figure legends as clear and stand-alone as possible, and consider specifying that the figure depicts predicted population dynamics according to SB2.

We apologize, this should have read "reference scenario". We have revised this caption for clarity.

> Figure 4: Daily time series of model dynamics for buffer sector, showing three scenarios (color): no intervention, adult sampling only, and container sampling only (ensemble median of means + 95% CI). In scenario 3, container sampling was not conducted during spray periods. See Figure 3 for additional details.

»Please provide more information about "the buffer sector" to make figure legends as clear and stand-alone as possible. In this case, you could consider referring to Figure 1 for clarity. Also please explain what additional details can be found in Figure 3.

We have revised this caption for clarity.

> Figure 5: Daily time series of model dynamics (ensemble median of means + 95% CI), comparing different spray efficacies (color) between sectors (rows within each panel). A, Adults/house. B, Proportion nulliparous females. See Figure 3 for additional details.

»consider adding additional details (e.g., Skeeter Buster 2) so figure legend stands alone; Also please explain what additional details can be found in Figure 3.

We have revised this caption for clarity.

> Figure 6: Simulated survey results. F

> consider adding detail to 'survey results', e.g., life stage sampled or expected

We have revised this caption for clarity.

> Figure 8: Detail of weekly mean AA/HSE within each sector (row) throughout experimental spraying, comparing empirical and simulated survey results (color). Simulation results show ensemble median + 95% PI. Vertical dashed lines shows approximate timing of spray cycles; during each cycle, a spray attempt was made at every house.

»please spell out AA/HSE in figure legend. Note spelling error 'shows'

Thank you, fixed.

> Figure 9: Comparison of simulated Ae. aegypti adults sampled per house (AA/HSE). AA/HSE within sector (row) among different spray efficacies. See Figure 3 and Figure 6 for additional details.

»consider adding detail to explain S-2013 and L-2014 in figure legend.

Thank you. We have added the per-year default spray efficacy to this caption, which offers important context.

Discussion.

> Our model explicitly tracks food resources within containers, but unfortunately these dynamics (and the resulting impact on larvae) are poorly characterized in natural settings. *»This point is not clear from the Methods, which state "and larval development depends on weather and food availability." How are food resources tracked within containers in the model? Please clarify either here or in Methods section.*

We have revised the *Simulation Overview* section (see above) to better highlight these dynamics, to note SB2's continuity in this regard with SB, and to better indicate the relevant literature surrounding SB formulation and parameterization.
