# Supplementary material for: A critical assessment of the detailed Aedes aegypti simulation model Skeeter Buster 2 using field experiments of indoor insecticidal control in Iquitos, Peru
Source: PLoS Negl Trop Dis. 2022 Dec 22;16(12):e0010863. doi: 10.1371/journal.pntd.0010863 (PMC9778528; doi:10.1371/journal.pntd.0010863)
Supplement: Attachment — Submitted filename: gunning-response.2022-09-23.docx [file pntd.0010863.s012.docx]

Dear editorial office,

We have edited author information and captions for Figs 3-4 per the editor’s requests.

Best,

Christian Gunning
